# Rapid detection of Golgi protein 73 by MAGLUMI chemiluminescent immunoassay and the clinical value to liver fibrosis/cirrhosis patients with chronic liver disease

**DOI:** 10.3389/fcimb.2025.1576045

**Published:** 2025-10-16

**Authors:** Lu Wang, Wen Dai, Jie Rao, Yichen Wang, Kun Liu, Xing Li, Hua Wang, Jiahuan Ye, Zhonggang Fang, Xin Zheng

**Affiliations:** ^1^ Department of Infectious Diseases, Union Hospital, Tongji Medical College, Huazhong University of Science and Technology, Wuhan, China; ^2^ Joint International Laboratory of Infection and Immunity, Huazhong University of Science and Technology, Wuhan, China; ^3^ Department of Clinical Laboratory, Institute of Translational Medicine, Renmin Hospital of Wuhan University, Wuhan, Hubei, China; ^4^ Research & Development Department, Shenzhen New Industries Biomedical Engineering, Shenzhen, Guangdong, China

**Keywords:** GP73, liver fibrosis, immunoassay, performance evaluation, diagnosis

## Abstract

**Objective:**

To investigate the diagnostic value of MAGLUMI chemiluminescent immunoassay (CLIA) for detecting Golgi protein 73 (GP73) in patients with chronic liver disease.

**Methods:**

A total of 212 patients with chronic liver disease were selected as the research subjects. METAVIR pathological staging was performed according to LSM values, and GP73 levels were detected by CLIA. Spearman analysis was used to analyze the correlation between GP73, LSM and METAVIR staging. The diagnostic efficacy of GP73 was analyzed using the ROC curve based on METAVIR staging.

**Results:**

The enrolled patients included 37 patients in F0/F1, 80 patients in F2, 61 patients in F3, and 34 patients in F4. There were significant differences in GP73 levels in each stage (*p*<0.001 for all stages). Spearman correlation analysis showed that GP73 levels were positively correlated with LSM and METAVIR stages. The AUC of GP73 in diagnosing significant liver fibrosis (F≥2), advanced liver fibrosis (F≥3), and cirrhosis (F=4) were 0.78 (95% CI: 0.72~0.84, *p*<0.0001), 0.83 (95% CI: 0.75~0.89, *p*<0.0001), 0.90 (95% CI: 0.80~0.96, *p*<0.0001), respectively.

**Conclusion:**

GP73 detected by CLIA was positively correlated with liver fibrosis stage and LSM, and had important clinical value in diagnosis of liver fibrosis and cirrhosis in patients with chronic liver disease.

## 1 Introduction

Liver fibrosis is an important factor affecting the prognosis of patients with chronic liver disease and a key step in the development of various chronic liver diseases to cirrhosis and hepatocellular carcinoma (HCC) (Tao et al., 2023). It refers to the diffuse excessive deposition and abnormal distribution of liver extracellular matrix (i.e. collagen, glycoproteins, proteoglycans, etc.) (Lee et al., 2015) and is the pathological result of various chronic liver diseases (Yang et al., 2020). Hepatic cell damage, inflammatory response and cell apoptosis caused by various reasons can induce liver fibrosis (Tao et al., 2023). Although various treatments targeting the etiology, such as antiviral drugs and immunosuppressive therapy, are available for the treatment of liver fibrosis, the efficacy is limited (Tao et al., 2023). Therefore, it is particularly important to accurately and timely assess the level of liver fibrosis, tailor treatments to the individual characteristics (Trautwein et al., 2015; Cai et al., 2021).

Golgi protein 73 (GP73) is a transmembrane protein on Golgi apparatus, primarily expressed in epithelial cells of many human tissues but was almost undetectable in normal hepatocytes (Marrero et al., 2005). It is highly expressed in the hepatocytes of patients with various acute and chronic liver diseases (Xu et al., 2018; Qian et al., 2019). When liver damage occurs, the amino terminus of GP73 is cleaved, releasing the carboxyl terminus of GP73 into the blood (Cao et al., 2017; Yao et al., 2019). Many studies (Qiao et al., 2014; Dong et al., 2017; Liu et al., 2018; Chen et al., 2022; Loosen et al., 2022; Xu et al., 2022) have shown that GP73 is closely related to liver fibrosis and cirrhosis in chronic liver diseases such as chronic hepatitis B (CHB), chronic hepatitis C (CHC), non-alcoholic fatty liver disease (NAFLD), alcoholic liver disease (ALD) and autoimmune hepatitis (AH).

In most cases, GP73 is detected using enzyme-linked immunosorbent assay (ELISA). Snibe (Shenzhen, China) has recently developed a chemiluminescence immunoassay (CLIA) for GP73 and this assay is not yet on the market and is still in the experimental stage.

This study consisted of three parts. The first part was to test the analytical performance of this GP73 assay reagent, and the analyzed performances were as follows: recovery experiment, precision, limit of blank (LoB), limit of detection (LoD), linearity interval and Anti-interference ability. The second part was method comparison and the third part aimed to evaluate the clinical value of GP73 levels detected by CLIA in diagnosing liver fibrosis and cirrhosis in patients with chronic liver disease.

## 2 Materials and methods

### 2.1 Analytical performances

Tested sample:

GP73 antigen produced by Shenzhen New Industry Biomedical Engineering Co., Ltd (Snibe) with Purity ≥ 90% (SDS-PAGE). The original concentration is 1.994 mg/mL and was diluted to several concentrations for analytical performance testing.

GP73 determination:

Golgi protein 73 assay kit (CLIA) (Snibe). Six fully automatic CLIA analyzers produced by Snibe were used: Maglumi 4000 Plus (M4000P), Maglumi 2000 Plus (M2000P), Maglumi 800 (M800), Maglumi X3 (X3), Maglumi X6 (X6), Maglumi X8 (X8).

Recovery experiment:

The recovery experiment was conducted using all six analyzers and diluted tested samples. The recovery rate was used to evaluate the performance.

Precision:

The precision was evaluated for all six analyzers by within-lot precision and between-lot precision. For within-lot precision, one lot of assay was used to test each of the three precision reference samples for 10 replicates. For between-lot precision, three lots of assays were used to test each of the three precision reference samples for 10 replicates. The coefficient of variation (CV) was calculated as:


$$\text{CV}=\frac{\text{standard deviation}}{\text{Mean}}\times 100\%$$

Limit of blank (LoB):

The LoB was evaluated for all six analyzers by testing blank sample (PBS buffer for this study) for 20 replicates and measured by the concentration corresponding to the “ mean+2×standard deviation “ of the analyzers’ light generation (in RLU).

Limit of detection (LoD):

The LoD was evaluated for all six analyzers by testing five samples at 2.000 ng/mL for 5 replicates.

Linearity interval:

The linearity interval was evaluated for all six analyzers by testing the six diluted samples followed by the linear correlation analysis.

Interference:

Concentrated hemoglobin, fat emulsion and bilirubin solutions were separately added to the samples as interference samples, so that the concentrations of hemoglobin, fat emulsion and bilirubin in the interference samples reached 1000 mg/dL, 2000 mg/dL and 60 mg/dL, respectively. The detection deviation was calculated, and its influence on the concentration of GP73 was determined.

### 2.2 Method comparison

Comparative kit:

A total of 303 samples was tested using the GP73-CLIA method and the commercially available GP73-ELISA kit. The GP73-ELISA kit is produced by Beijing Hotgen Biotech Co., Ltd. (China). Its linear range is 50–500 ng/mL, with a sensitivity of ≤ 25 ng/mL and a intra-assay precision of ≤ 15 ng/mL.

Statistical analysis:

MedCalc Statistical Software version 19.2.6 (Belgium) was used to process and analyze the data. Spearman Correlation analysis was used to check the correlation between the comparative kit and the GP73 kit by Snibe. Bland-Altman analysis including the corresponding data behavior analysis (Homogeneity of Variance Test and proportional bias) of the difference percentage was used to test the agreements between the comparative kit and the GP73 kit by Snibe.

### 2.3 Clinical value

Reference interval:

A group of healthy individuals with normal liver function who have no history or current infection of hepatitis viruses as the reference population. Calculate the median of GP73 levels and use non-parametric methods to determine the 95^th^ reference range for overall samples.

Patients:

This study included 212 patients with chronic liver disease enrolled at Union Hospital Tongji Medical College Huazhong University of Science and Technology and Renmin Hospital of Wuhan University from September 2022 to May 2023. Briefly, the patients inclusion criteria included age greater than 18 years but below 80 years, diagnosis of chronic liver disease including CHB, CHC, NAFLD, ALD or AIH and the duration is more than six months, had liver transient elastography (TE) and met the consensus opinion on the non-invasive diagnosis of liver fibrosis using TE technology FibroScan in *Consensus on clinical application of transient elastography detecting liver fibrosis: a 2018 update* (Chinese Foundation for Hepatitis Prevention and Control et al., 2019) and *Consensus on the diagnosis and therapy of hepatic fibrosis (2019)* (Lu et al., 2019). Patients were excluded if they had the following: presence of other causes of liver disease, prior liver transplantation, incomplete data, obesity, intercostal space narrowing, and obvious ascites that cause TE test failure, treatment with liver-protecting and enzyme-lowering drugs such as glycyrrhizin and silibinin within one week, with a history of alcoholism^1^. All patients gave informed consent for this study and agreed to collect relevant clinical diagnostic background information of the patients involved in the above consensus. This study was approved by the ethics committees of Union Hospital Tongji Medical College Huazhong University of Science and Technology and Renmin Hospital of Wuhan University.

TE test:

FibroScan-502 Touch from ECHOSENS (France) was used for TE test to detect the liver stiffness measure (LSM) of the patients. The test was performed by an operator who has successfully performed more than 500 consecutive operations. During the test, after the operator confirms that the patient meets the FibroScan test criteria, the patient lies on his/her back and puts his/her right arm on his/her head to fully expose the right intercostal space. The operator strictly follows the operating procedures and holds the probe to the test area surrounded by the patient’s xiphoid process horizontal line, right axillary midline and the lower edge of the ribs for testing. The final effective LSM test requires that the same test point is successfully tested more than 10 times and the ratio of the interquartile difference of the test value to the median is less than 0.3.According to Consensus on clinical application of transient elastography detecting liver fibrosis: a 2018 update, the cut-off values for LSM of different etiologies in the studied patients were defined, as shown in **Table 1**. Based on LSM, the METAVIR fibrosis stage (F0/F1, F2, F3, F4) of these patient can be determined (Chinese Foundation for Hepatitis Prevention and Control et al., 2019).

**Table 1:** Cut-off values for LSM of different etiologies.

Variable	F0/F1	F2	F3	F4
CHB ALT< 5 × ULN	< 9.4	9.4 - 12.4	12.4 - 17.0	≥17.0
CHB normal ALT	< 6.0	6.0 - 9.0	9.0 - 12.0	≥12.0
CHC	< 8.8	8.8 - 9.3	9.3 - 14.6	≥14.6
NAFLD	< 9.0	9.0 - 11.0	11.0 - 15.0	≥15.0
ALD	< 9.5	9.5 - 12.5	12.5 - 20.0	≥20.0
AIH	< 9.6	9.6 - 10.7	10.7 - 16.0	≥16.0

The units for all values are kPa. The intervals of F2 and F3 stages are in the form [lower, upper). ALT, alanine transaminase; ULN, upper limit of normal.

GP73 determination:

The level of GP73 in serum was detected by the Golgi protein 73 assay kit (CLIA) produced by Shenzhen New Industry Biomedical Engineering Co., Ltd (Snibe). The instrument was the fully automatic CLIA analyzer MAGLUMI X8 produced by Snibe.

Statistical Analysis:

SPSS 20.0.0 (Chicago) software was used to process and analyze the data. Shapiro-Wilk test was applied to the quantitative data of this study for normality test. The normally distributed data variables were described as “mean±standard deviation” while the others were described as “median (quartile1 ~ quartile3)”. Mann-Whitney U test was used for inter-group comparison; the correlation between different variables was analyzed by Spearman Correlation Analysis. The area under receiver operating curve (AUC) was used to evaluate the diagnostic efficacy of serum GP73 for liver fibrosis/cirrhosis in the studied patients. *p*<0.05 was considered a statistically significant difference.

## 3 Results

### 3.1 Analytical performances

Recovery experiment:

The results of the recovery experiment is shown in **Supplementary Table 1**. The recovery rate of all six analyzers range from 94.37% to 101.38%, within ±10%.

Precision:

The within-lot precision results are shown in **Supplementary Table 2** and the between-lot precision results are shown in **Supplementary Table 3**. For within-lot precision, the CV ranged from 1.06% to 3.03%. For between-lot precision, the CV ranged from 0.99% to 3.09%. Both within-lot and between-lot precision was observed that at a satisfactory CV of 5%.

LoB:

PBS buffer was used as the blank sample. As shown in **Supplementary Table 4**, all six analyzers tested the blank sample with the concentration corresponding to the “ mean+2×standard deviation “ of the analyzers’ light generation less than 1 ng/mL.

LoD:

Each single test results of LoD test are shown in **Supplementary Table 5**. For all replicates test of each analyzers, and there were less than 3 ng/mL.

Linearity interval:

The results of linear correlation analysis are shown in **Supplementary Table 6** and **Supplementary Figure 1**. For all six analyzers, the coefficient R^2^ between actual concentration and the theoretical concentration are over 0.99, which means all the different analyzer have a good linearity. The linearity interval could be defined as [4, 1000] ng/mL.

Anti-interference ability:

The anti-interference results show that when the samples contains up to 1000 mg/dL of hemoglobin, 2000 mg/dL of lipid emulsion and 60 mg/dL of bilirubin respectively, the detection variation of the GP73-CLIA kit are -3.5%, -4.1% and 3.6%, respectively, which are all within 5%.

### 3.2 Method comparison

In the Method Comparison study, the GP73-CLIA reagent and the commercially available GP73-ELISA kit were used for the evaluation. The parameters of the two reagents are shown in **Supplementary Table 7**.

**Figure 1:**
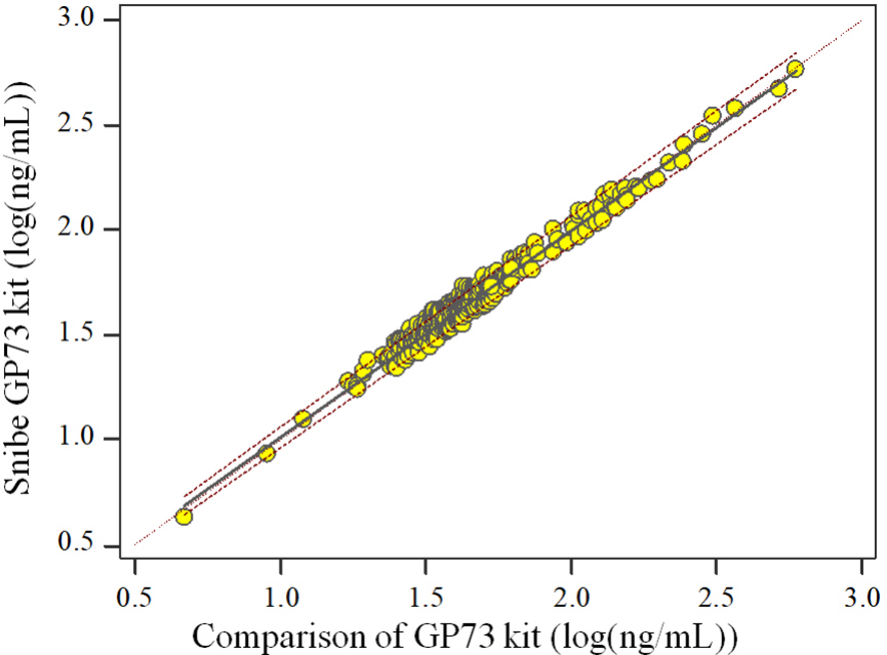
Scatter plot of the two kits.

The Spearman Correlation analysis shows the quantity results of the two kits are positively correlated. The correlation coefficient *r_s_* is 0.997 (*p<*0.05) and the regression equation is y=1.096+0.982 x , Since the majority of the sample detection values were<200 ng/mL, we presented the scatter plot of the log-transformed detection values to facilitate a clearer interpretation of the data (**Figure 1**).

**Figure 2:**
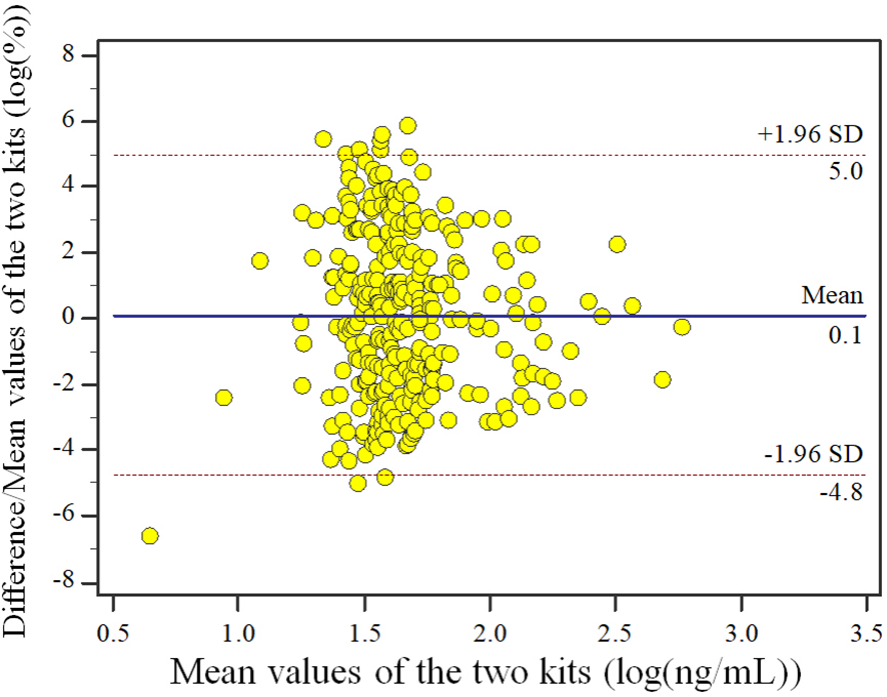
Bland-Altman plot of the two kits.

The results of data behavior analysis showed that the percentage of difference/mean between the assessment reagent and the measurement confirmation comparison reagent for clinical samples had no proportional bias and equal variance. The upper Limits of Agreement (LoA) bound is 18.36% (95%CI: 16.60%~20.13%) and the lower LoA bound is -17.62% (95%CI: -19.39%~-15.86%). Of all samples (n=303), there are 4 samples locate outside the 95% LoA interval. Similarly, we presented the Bland-Altman plot of the log-transformed detection values to facilitate a clearer interpretation of the data (**Figure 2**).

### 3.3 Clinical value

General Information:

A total of 229 healthy individuals participated in the reference interval study, aged 19 to 30 (n=47), 31 to 40 (n=44), 41 to 50 (n=48), 51 to 60 (n=47), >60 (n=43) years old, including 108 males and 128 females. Our research results indicate that the median value of GP73 in the overall healthy population is 22.1 ng/mL, and the upper limit of the 95^th^ reference range is 37.35 ng/mL (**Supplementary Table 8**).

This disease study included 212 patients with chronic liver disease, including 136 males and 76 females, aged 21 to 80 years old. Among the included patients, there were 145 patients with CHB, 11 patients with CHC, 26 patients with NAFLD, 14 patients with ALD, and 16 patients with AIH. The Characteristics of the studied patients is shown in **Table 2**. The test values of the reference interval population and the disease group showed a statistically significant difference (p<0.05).

**Table 2:** Patients' characteristics.

Variable	F0/F1	F2	F3	F4
Sex, n (%)				
Male	23 (62.2%)	53 (66.3%)	36 (59.0%)	24 (70.6%)
Female	14 (37.8%)	27 (33.8%)	25 (41.0%)	10 (29.4%)
Age mean(±SD)	44.32(±10.29)	48.18(±12.56)	47.30(±12.65)	52.65 (±12.86)
GP73* median(IQR)	34.30 (25.10-39.22)	41.37 (34.36-52.26)	49.70 (35.81-67.37)	80.42 (42.92-121.03)

*Indicates the variable does not conform to normal distribution.

**Figure 3:**
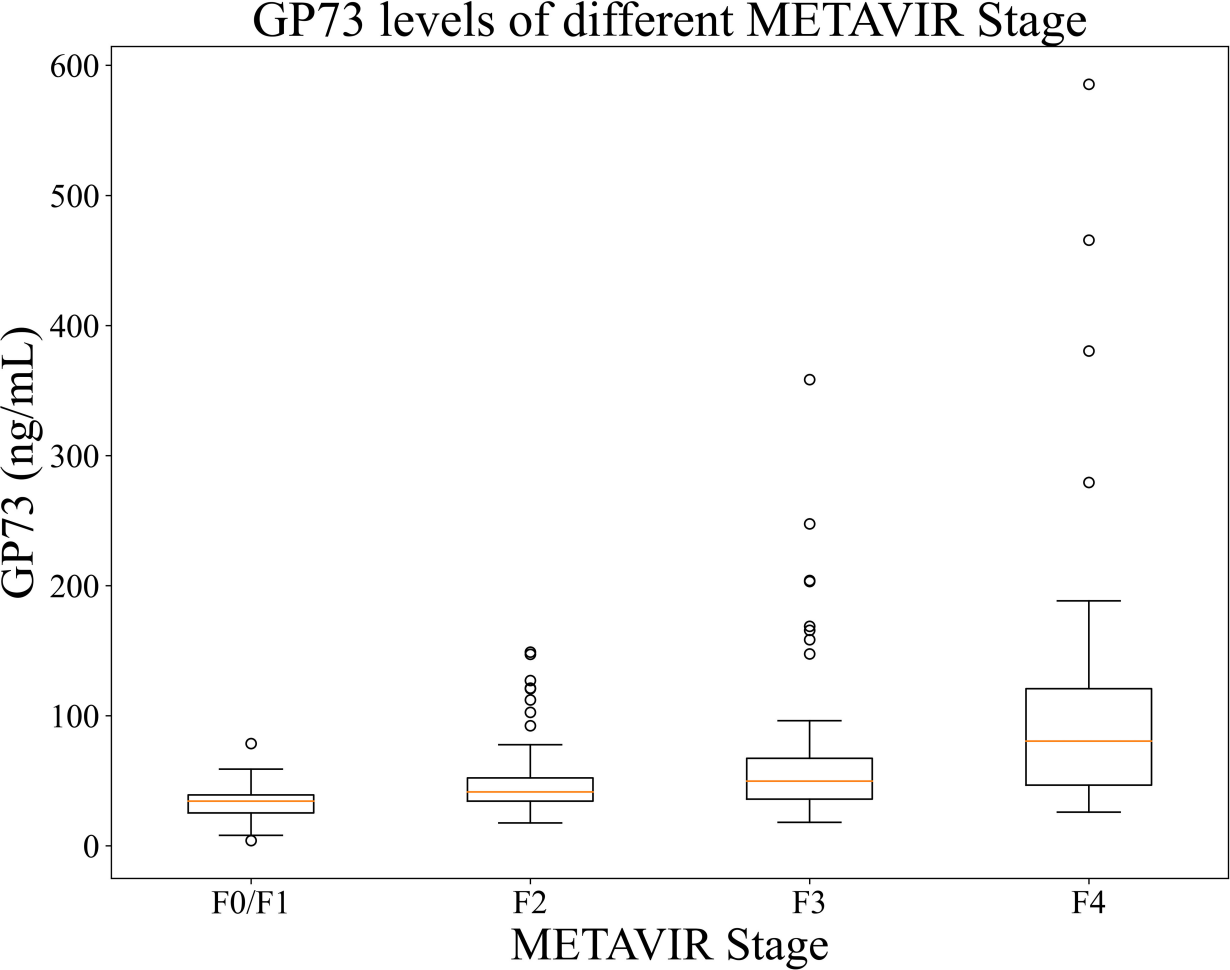
GP73 levels of patients in each stages. The between-group difference was compared using Mann-Whitney U test, p <0.001 for each two groups.

Comparison of GP73 levels in different METAVIR stages:

The mean GP73 level in different liver fibrosis stages F0/F1 (n=37 cases), F2 (n=80 cases), F3 (n=61 cases), and F4 (n=34 cases) were 34.30 (95%CI: 26.47-36.32) ng/mL, 41.37 (95%CI: 38.25-44.82) ng/mL, 49.70 (95%CI: 41.73-57.55) ng/mL, and 80.42 (95%CI: 63.37-105.94) ng/mL, respectively. As METAVIR stage of liver fibrosis increased, the GP73 level also increased, and the differences were statistically significant (*p*<0.001), as shown in **Figure 3**.

Correlation of GP73 with LSM and METAVIR stages:

GP73 level was positively correlated with LSM and METAVIR stage of liver fibrosis: the Spearman correlation coefficient with LSM was r_s_=0.51 (*p*<0.001); the Spearman correlation coefficient calculated after assigning METAVIR stage of liver fibrosis was r_s_=0.47 (*p*<0.001), as shown in **Table 3**.

**Table 3:** Correlation between GP73 level, LSM and METAVIR stages.

Variable	Correlation coefficient	*P* value
LSM	0.51 (95% CI: 0.40-0.60)	<0.0001
METAVIR stages	0.47 (95% CI: 0.36-0.57)	<0.0001

The correlation was analyzed by Spearman Correlation Analysis.

**Figure 4:**
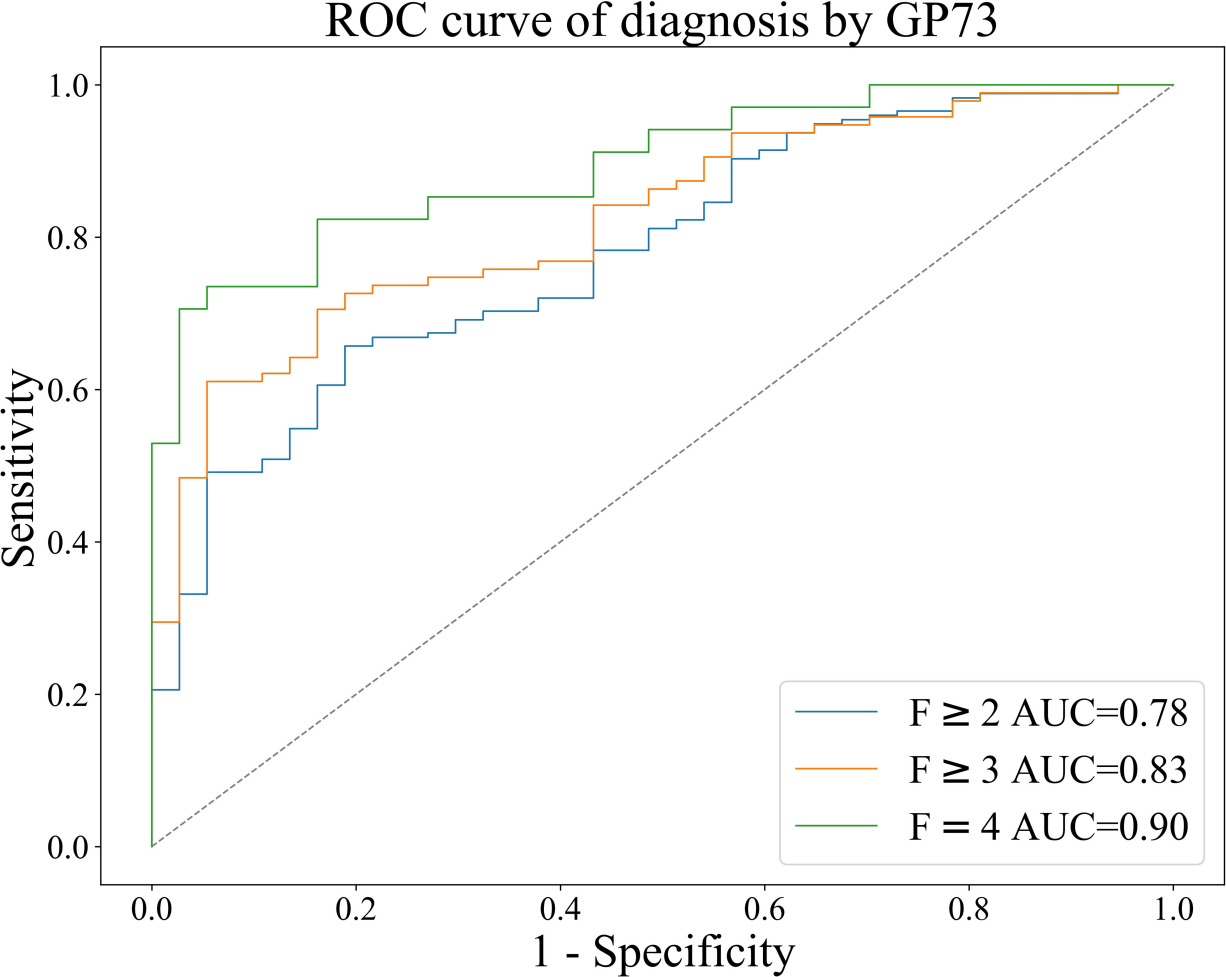
ROC curve of GP73 for the diagnosis of liver fibrosis in patients with chronic liver disease.

Diagnostic efficacy of GP73 and METAVIR stages:

Based on the METAVIR staging system, AUC was used to evaluate the diagnostic efficacy of GP73-CLIA for liver fibrosis in patients with chronic liver disease. **Figure 4** shows the receiver operating curve (ROC) of GP73 in diagnosing different fibrosis stages.

The results were as follows: the AUCs of GP73 in diagnosing significant liver fibrosis (F≥2), advanced liver fibrosis (F≥3), and early cirrhosis (F=4) are 0.78, 0.83, and 0.90, respectively (**Table 4**). Youden index (Youden, 1950) was used to determine the optimal cut-off value, and its sensitivity and specificity were 39.70 ng/mL (66.86%, 78.38%), 40.80 ng/mL (71.57%, 81.08%), 42.04 ng/mL (82.35%, 83.78%), as shown in **Table 4**.

**Table 4:** Diagnostic efficacy of GP73 for liver fibrosis in patients with chronic liver disease.

Stage	AUC (95%CI)	*P* value	Cut-Off	Sensitivity	Specificity
F≥2	0.78 (0.72-0.84)	<0.0001	39.70	66.86%	78.38%
F≥3	0.83 (0.75-0.89)	<0.0001	40.80	71.57%	81.08%
F=4	0.90 (0.80-0.96)	<0.0001	42.04	82.35%	83.78%

## 4 Discussion

Liver biopsy is the gold standard for diagnosing fibrosis. However, liver biopsy is an invasive and may be accompanied by complications which limit its universal application (Sumida et al., 2014). Serum marker tests and imaging examination techniques make up for the difficulties in dynamic monitoring of liver fibrosis due to their good tolerability, acceptability and repeatability. Transient elastography is the most commonly used imaging technique for non-invasive diagnosis of liver fibrosis all over the world (Singh et al., 2017; European Association for Study of Liver, 2021). In China, it is also an important method for evaluating the degree of liver fibrosis in patients with chronic liver diseases such as CHB, CHC, NAFLD, ALD and AIH (Lu et al., 2019). At present, there is still a lack of a single serum marker with high specificity for diagnosing liver fibrosis. Clinically, the diagnostic value of serum markers for liver fibrosis mainly depends on combined detection of hematological non-invasive diagnostic models, such as FIB-4 and APRI, but they all have shortcomings including but not limited to single applicable disease, unstable diagnostic performance, limited applicable population (Liu et al., 2018; Chen et al., 2022; Loosen et al., 2022; Xu et al., 2022). Therefore, it is necessary to explore new serum markers that can be effectively and widely used in the clinical diagnosis of liver fibrosis/cirrhosis.

GP73 is a transmembrane protein on the Golgi apparatus. It was first discovered by Kladney et al. in 2000 (Kladney et al., 2000). Subsequent studies by Kladney et al. found that GP73 was significantly higher in patients with CHB, CHC, ALD and AIH than in normal people (Kladney et al., 2002). In recent years, many studies have shown that GP73 is closely related to liver fibrosis and cirrhosis in patients with chronic liver disease. Liu et al. found that the correlation coefficient between GP73 and liver fibrosis in children with chronic liver diseases, including genetic diseases, viral hepatitis, autoimmune hepatitis, and congenital malformations was 0.338 (*p*<0.001) (Kladney et al., 2000). Yao et al. found that the correlation coefficient between GP73 and liver fibrosis in patients with HBV was 0.434 (*p*<0.001) (Yao MingJie. et al., 2018). Li et al. found that the correlation coefficient between serum GP73 and liver fibrosis at different stages of NAFLD was 0.436 (*p*<0.001) (Li et al., 2021).

As for the diagnostic efficacy of GP73 for liver fibrosis and cirrhosis, Yao et al. found the AUC of GP73 diagnosing significant liver fibrosis (F≥2), advanced liver fibrosis (F≥3), and early cirrhosis (F=4) were 0.772, 0.854 and 0.890, respectively, for virus hepatitis, 0.855, 0.852 and 0.880, respectively, for AIH, and 0.897, 0.935 and 0.960, respectively, for NAFLD (Yao Mingjie. et al., 2018). In addition, the patent study by Lu et al. found that the AUC, sensitivity and specificity of GP73 for the diagnosis of liver fibrosis F≥2, F≥3 and F=4 were 0.828 (64.92%, 90.68%), 0.895 (80.66%, 86.49%), 0.933 (82.61%, 91.24%), respectively, proving that GP73 can also distinguish between significant liver fibrosis and advanced liver fibrosis in patients with liver fibrosis not caused by hepatitis B virus infection (Lu et al., 2017). Moreover, the study by An et al. further confirmed that GP73 is also suitable for the diagnosis of different degrees of liver fibrosis in patients with chronic liver diseases other than CHB (An et al., 2023).

The above studies have demonstrated the diagnostic value of GP73 for liver fibrosis in patients with chronic liver disease, but all of them were conducted using ELISA. When transitioning from academic research to large-scale clinical applications, ELISA demonstrates poor cost-effectiveness. In contrast, the CLIA reagent developed in this study offers high-throughput and automated capabilities. The GP73-CLIA assay completes the entire testing process-from sample loading to result reporting-in approximately 20 minutes per specimen, with X8 analyzer capable of processing 300 samples per batch. Conversely, ELISA requires manual, stepwise operations with a turnaround time exceeding 2 hours per sample. For calibration, each CLIA reagent batch contains an integrated standard curve, eliminating the additional calibration steps required for ELISA. Furthermore, conventional ELISA carries inherent limitations, particularly the elevated contamination risk associated with manual handling. Regarding analytical performance, our GP73-CLIA reagent demonstrates significant advantages over existing ELISA methods, as detailed in **Supplementary Table 7**. Compared to manufacturers’ claimed analytical performance for commercially available ELISA kits, GP73-CLIA shows better performance in linear range (4–1000 ng/mL *vs* 50–500 ng/mL), intra-assay precision (CV, ≤10% *vs ≤*15%) and sensitivity (≤1 ng/mL *vs ≤*25 ng/mL).

This study included 212 patients with chronic liver disease caused by different causes who underwent FibroScan examination in two research centers. METAVIR staging was performed according to the consensus opinion on non-invasive diagnosis of liver fibrosis using transient elastography technology FibroScan. Serum GP73 levels of patients at each stage were detected by GP73-CLIA. It was found that with the increase of METAVIR staging of patients with chronic liver disease, the degree of liver fibrosis worsened and the GP73 level gradually increased (*p*<0.01). Subsequently, the Spearman correlation analysis showed that GP73 was positively correlated with LSM and METAVIR stage of liver fibrosis (r_s_=0.51, *p*<0.0001; r_s_=0.47, *p*<0.0001), and the degree of correlation was consistent with the results of studies by and Li et al. (2021) and Yao et al (Yao MingJie. et al., 2018). Finally, the diagnostic efficacy of GP73 in this study is basically consistent with the results of Yao Mingjie. et al. (2018) and Lu et al (Lu et al., 2017). Therefore, GP73 can be considered for auxiliary diagnosis and staging prediction of liver fibrosis. We think it can be serve as a screening method for high-risk populations and for stratifying patients. For samples exceeding the reference threshold (indicating potential fibrosis), particularly those with significantly elevated values, we recommend secondary methods (such as transient elastography or liver biopsy) to assess the liver fibrosis condition of the potential patient and to mitigate potential risks of disease underestimation.

The benefits of our GP73-CLIA method can be summarized into three aspects. One, health economic benefits: GP73-CLIA has the advantage of high throughput. While improving the detection efficiency, it significantly reduces labor costs, making it suitable for universal screening of high-risk populations such as patients with chronic liver disease, and beneficial for stratified management. Through early detection, liver fibrosis can be detected early and treatment costs for advanced liver cirrhosis can be saved. Two, excellent analytical performance: The linear range of GP73-CLIA is superior to other commercially available ELISA kit. For example, in the F4-stage liver fibrosis case in our study, the maximum concentration reached 585.444 ng/mL. Thus, the wide linear interval cover populations with extremely high concentrations, thereby minimizing manual dilution errors. In terms of interference resistance, the study found compatibility with complex samples, such as the hyperlipidemia commonly seen in liver disease patients, with results indicating no interference. Three, clinical performance: Our findings indicate that GP73 testing alone may aid in auxiliary diagnosis of liver fibrosis and the prediction for fibrosis staging. However, as for the shortcomings, lack of histological diagnosis of liver fibrosis stages comes first as liver biopsy is the gold standard. Patients with histological diagnosis would be included in further studies. Moreover, it has been reported that serum GP73 level could be influenced by the hepatic inflammation activity, This study included patients with chronic liver diseases undergoing regular follow-up and did not analyze the correlation between GPP3 and liver inflammation markers (ALT, AST, *etc.*). This will need to be further improved in the future. Besides, the small clinical sample size and not enough samples for different etiologies and health control should also be ameliorated. Finally, the diagnostic efficacy of GP73 combined with other non-invasive laboratory serum markers of liver fibrosis and imaging examinations need further in-depth research.

## 5 Conclusion

The CLIA GP73 assay by Snibe has satisfactory analytical performances and GP73 detected by CLIA is positively correlated with liver fibrosis stage and LSM, and has important clinical value in diagnosis and staging of liver fibrosis and cirrhosis in patients with chronic liver disease.

## Data Availability

The raw data supporting the conclusions of this article will be made available by the authors, without undue reservation.

## Footnotes

1 Long-term drinking history, generally more than 5 years, equivalent to 40 g/day for men and 20 g/day for women, or heavy drinking history within 2 weeks, equivalent to 80 g/day[Chinese Medical Association et al., 2020].
