# Supplementary material for: Rapid detection of Golgi protein 73 by MAGLUMI chemiluminescent immunoassay and the clinical value to liver fibrosis/cirrhosis patients with chronic liver disease
Source: Front Cell Infect Microbiol. 2025 Oct 16;15:1576045. doi: 10.3389/fcimb.2025.1576045 (PMC12571778; doi:10.3389/fcimb.2025.1576045)
Supplement: Supplementary file 1 [file DataSheet1.docx]

## Supplemental Materials

Supplemental Table 1. Recovery experiment.

|  | Theoretical concentration  (ng/mL) | Experimental concentration  (ng/mL) | Recovery rate |
| --- | --- | --- | --- |
| M4000P | 226.30 | 219.35 | 96.93% |
| M2000P | 223.00 | 212.00 | 95.07% |
| M800 | 214.70 | 214.80 | 100.05% |
| X3 | 218.00 | 221.00 | 101.38% |
| X6 | 206.00 | 194.40 | 94.37% |
| X8 | 217.00 | 216.60 | 99.82% |

Supplemental Table 2. Within-lot precision.

|  | Concentration 1  (ng/mL) | Concentration 2  (ng/mL) | Concentration 3  (ng/mL) |
| --- | --- | --- | --- |
| M4000P |  |  |  |
| Mean | 26.90 | 52.35 | 206.28 |
| SD | 0.44 | 0.61 | 3.04 |
| CV, % | 1.63 | 1.17 | 1.47 |
| M2000P |  |  |  |
| Mean | 26.97 | 48.87 | 172.80 |
| SD | 0.69 | 0.69 | 3.36 |
| CV, % | 2.55 | 1.42 | 1.94 |
| M800 |  |  |  |
| Mean | 26.16 | 48.10 | 171.20 |
| SD | 0.45 | 0.93 | 3.39 |
| CV, % | 1.71 | 1.93 | 1.98 |
| X3 |  |  |  |
| Mean | 27.46 | 50.14 | 176.60 |
| SD | 0.29 | 1.04 | 1.90 |
| CV, % | 1.06 | 2.08 | 1.07 |
| X6 |  |  |  |
| Mean | 27.81 | 50.45 | 179.50 |
| SD | 0.98 | 1.13 | 4.93 |
| CV, % | 3.54 | 2.24 | 2.74 |
| X8 |  |  |  |
| Mean | 27.01 | 52.25 | 181.90 |
| SD | 0.82 | 1.39 | 5.22 |
| CV, % | 3.03 | 2.66 | 2.87 |

SD: standard deviation; CV: coefficient of variation.

Supplemental Table 3. Between-lot precision.

|  | Concentration 1  (ng/mL) | Concentration 2  (ng/mL) | Concentration 3  (ng/mL) |
| --- | --- | --- | --- |
| M4000P |  |  |  |
| Mean | 26.37 | 52.54 | 207.35 |
| SD | 0.30 | 0.85 | 4.33 |
| CV, % | 1.15 | 1.62 | 2.09 |
| M2000P |  |  |  |
| Mean | 26.50 | 48.88 | 175.75 |
| SD | 0.26 | 0.56 | 3.71 |
| CV, % | 0.99 | 1.14 | 2.11 |
| M800 |  |  |  |
| Mean | 26.54 | 49.10 | 172.15 |
| SD | 0.30 | 0.52 | 3.62 |
| CV, % | 1.12 | 1.06 | 2.10 |
| X3 |  |  |  |
| Mean | 26.57 | 52.48 | 177.01 |
| SD | 0.30 | 0.92 | 3.60 |
| CV, % | 1.13 | 1.75 | 2.03 |
| X6 |  |  |  |
| Mean | 26.51 | 52.57 | 182.83 |
| SD | 0.32 | 0.89 | 2.78 |
| CV, % | 1.22 | 1.69 | 1.52 |
| X8 |  |  |  |
| Mean | 26.54 | 52.64 | 182.85 |
| SD | 0.59 | 1.69 | 5.66 |
| CV, % | 2.23 | 3.21 | 3.09 |

SD: standard deviation; CV: coefficient of variation.

Supplemental Table 4. Limit of blank test.

|  | Mean  (RLU) | SD  (RLU) | M+2SD  (RLU) | Concentration  corresponding to M+2SD  (ng/mL) |
| --- | --- | --- | --- | --- |
| M4000P | 890 | 219.45 | 1329.00 | 0.677 |
| M2000P | 968 | 158.36 | 1284.72 | 0.682 |
| M800 | 699 | 82.95 | 865.30 | 0.609 |
| X3 | 955 | 226.03 | 1407.21 | 0.796 |
| X6 | 683 | 40.29 | 763.48 | 0.677 |
| X8 | 805 | 70.77 | 946.09 | 0.535 |

SD: standard deviation; RLU: relative light unit.

Supplemental Table 5. Limit of detection test.

|  | M4000P | M2000P | M800 | X3 | X6 | X8 |
| --- | --- | --- | --- | --- | --- | --- |
| Sample  (2.000ng/mL) | Concentration  ng/mL | Concentration  ng/mL | Concentration  ng/mL | Concentration  ng/mL | Concentration  ng/mL | Concentration  ng/mL |
| Sample 1 | 1.89 | 1.98 | 1.72 | 2.12 | 1.75 | 1.94 |
|  | 2.13 | 2.07 | 1.85 | 2.04 | 2.10 | 2.12 |
|  | 2.12 | 2.00 | 2.02 | 2.21 | 1.94 | 1.85 |
|  | 2.17 | 2.07 | 1.85 | 2.10 | 1.94 | 1.85 |
|  | 2.25 | 1.91 | 1.70 | 2.17 | 1.88 | 1.89 |
| Sample 2 | 2.22 | 1.88 | 1.93 | 2.07 | 1.88 | 2.02 |
|  | 1.98 | 1.97 | 1.76 | 2.16 | 1.99 | 1.89 |
|  | 2.10 | 2.05 | 1.67 | 2.16 | 2.01 | 1.94 |
|  | 2.06 | 1.94 | 1.98 | 2.25 | 1.94 | 1.88 |
|  | 2.12 | 2.00 | 2.03 | 2.15 | 1.91 | 1.85 |
| Sample 3 | 2.17 | 2.10 | 2.04 | 2.09 | 1.92 | 2.07 |
|  | 2.00 | 2.05 | 1.83 | 2.16 | 2.04 | 1.72 |
|  | 1.99 | 1.92 | 1.75 | 2.15 | 2.01 | 2.03 |
|  | 1.93 | 1.96 | 2.66 | 2.11 | 1.98 | 1.90 |
|  | 1.97 | 2.08 | 1.95 | 2.06 | 1.87 | 1.93 |
| Sample 4 | 2.00 | 2.14 | 1.92 | 2.16 | 1.88 | 2.05 |
|  | 2.01 | 1.94 | 1.80 | 2.22 | 2.05 | 1.73 |
|  | 1.91 | 2.02 | 1.83 | 2.12 | 1.91 | 2.08 |
|  | 1.99 | 2.20 | 1.96 | 2.16 | 1.98 | 1.95 |
|  | 2.04 | 2.12 | 1.80 | 2.14 | 1.96 | 1.88 |
| Sample 5 | 1.88 | 2.05 | 1.93 | 2.12 | 1.77 | 2.15 |
|  | 1.92 | 2.02 | 1.79 | 2.13 | 1.96 | 1.94 |
|  | 2.01 | 2.01 | 1.94 | 2.14 | 2.10 | 2.11 |
|  | 2.09 | 1.91 | 1.83 | 2.09 | 1.99 | 1.86 |
|  | 1.99 | 1.96 | 1.74 | 2.18 | 1.86 | 1.91 |

Supplemental Table 6. Linearity interval test.

|  | Mean tested value (ng/mL) | | | | | |
| --- | --- | --- | --- | --- | --- | --- |
|  | M4000P | M2000P | M800 | X3 | X6 | X8 |
| Sample 1 (4 ng/mL) | 4.06 | 4.3 | 4.55 | 4.21 | 3.15 | 5 |
| Sample 2 (40.20 ng/mL) | 38.5 | 38.4 | 40.2 | 39.7 | 40.3 | 41.0 |
| Sample 3 (100.60 ng/mL) | 96.7 | 96.5 | 97.3 | 99.2 | 98.4 | 100.3 |
| Sample 4 (203.20 ng/mL) | 164 | 161 | 172 | 166 | 168 | 175 |
| Sample 5 (402.40 ng/mL) | 333 | 322 | 333 | 328 | 346 | 350 |
| Sample 6 (601.60 ng/mL) | 508 | 491 | 527 | 500 | 536 | 542 |
| Sample 7 (800.80 ng/mL) | 714 | 657 | 718 | 711 | 783 | 789 |
| Sample 8 (1000 ng/mL) | 943 | 908 | 989 | 954 | 1000 | 966 |

Supplemental Table 7. Comparison of parameters between CLIA and ELISA.

|  | GP73-CLIA | GP73-ELISA |
| --- | --- | --- |
| linear range (ng/mL) | 4-1000 | 50-500 |
| sample volume (μL) | 10 | 20 |
| precision (%) | ≤10 | ≤15 |
| sensitivity (ng/mL) | ≤1 | ≤25 |
| detection time | 20 min | > 2 hour |

The parameters of GP73-ELISA were derived from the manufacturer's (Beijing Hotgen Biotech Co., Ltd.) manual.

Supplemental Table 8. Reference intervals (RIs) for GP73-CLIA in healthy individuals.

| Age (years) | N | median (ng/mL) | 95th RIs (ng/mL) |
| --- | --- | --- | --- |
| 19-30 | 47 | 21.10 | ≤ 36.32 |
| 31-40 | 44 | 21.25 | ≤ 34.13 |
| 41-50 | 48 | 18.35 | ≤ 36.32 |
| 51-60 | 47 | 22.20 | ≤ 36.50 |
| >60 | 43 | 31.60 | ≤ 44.86 |
| All | 229 | 25.90 | ≤ 37.35 |


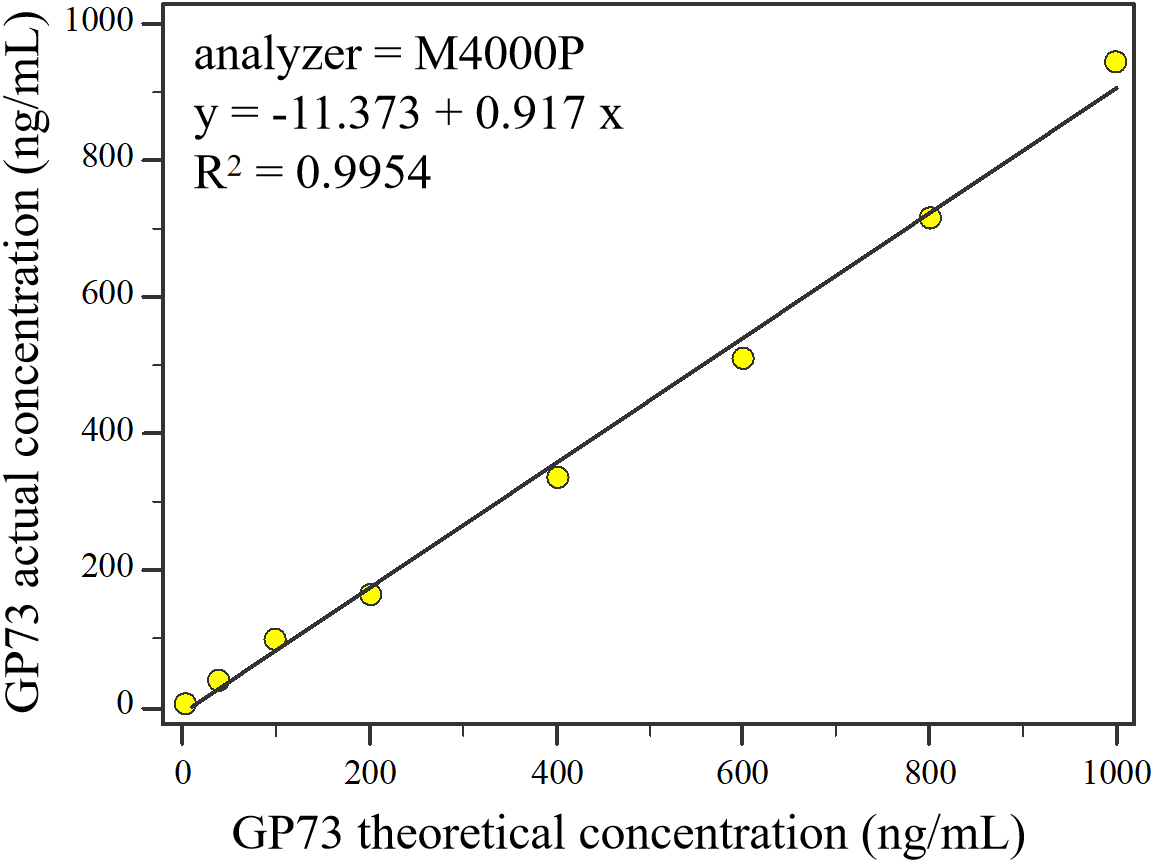

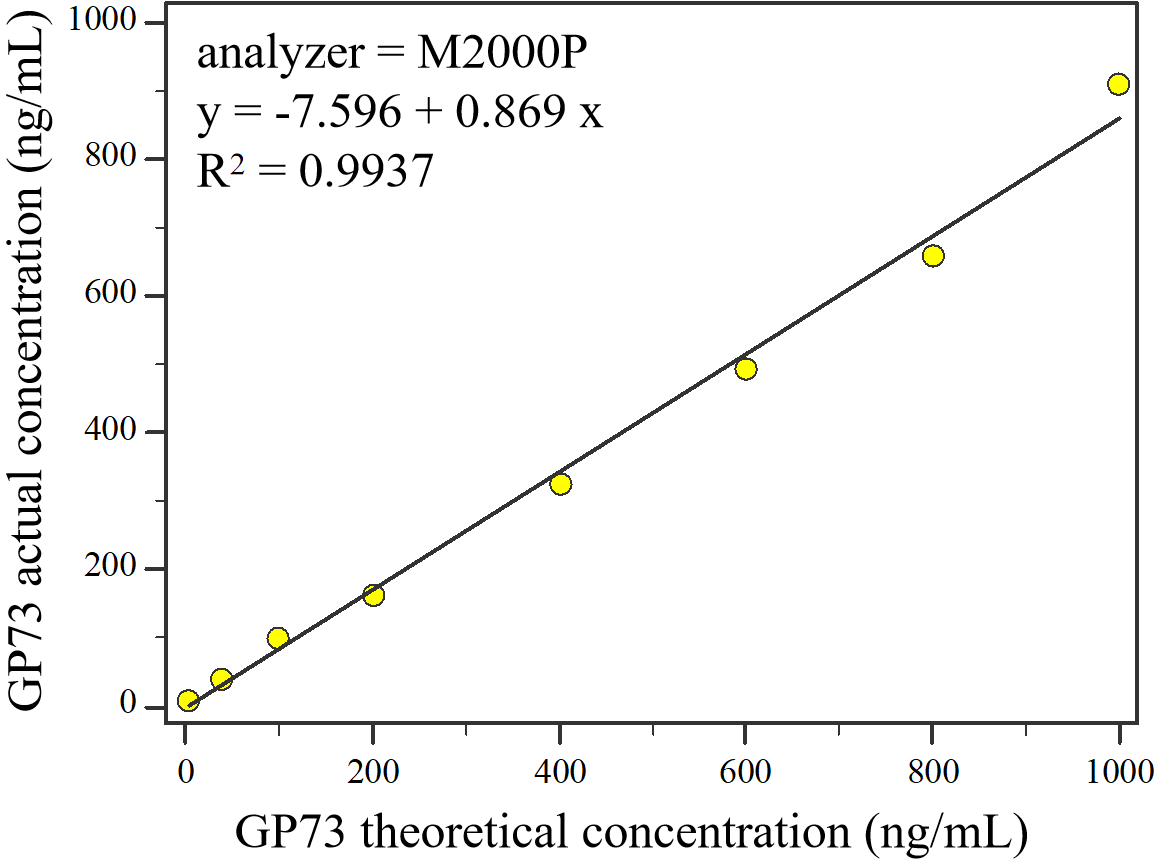


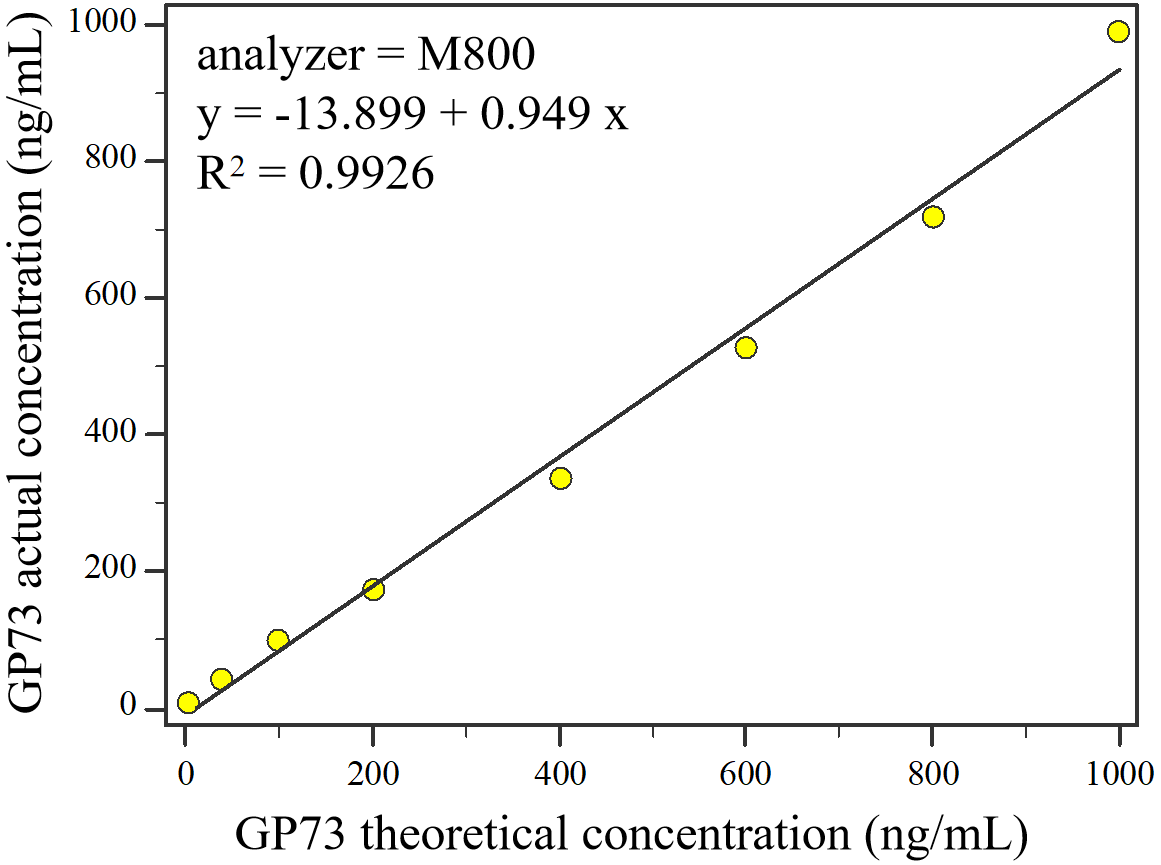

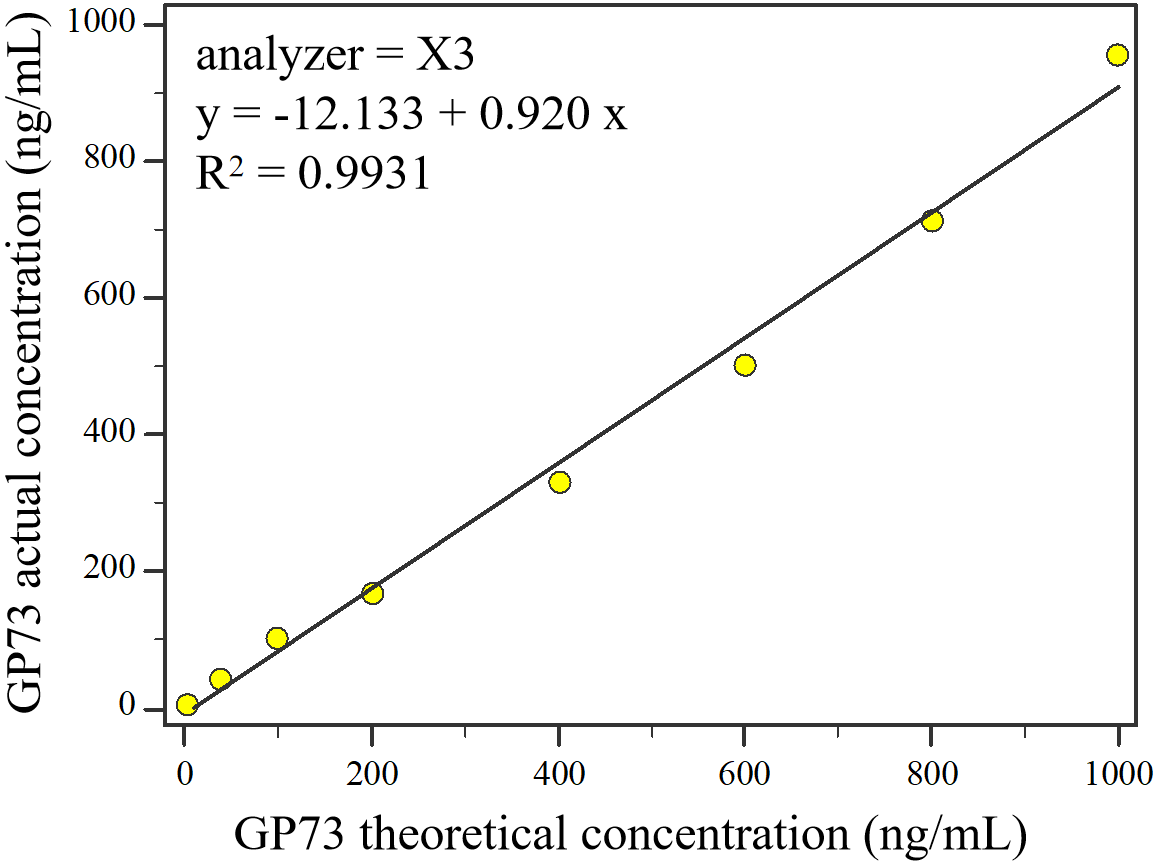


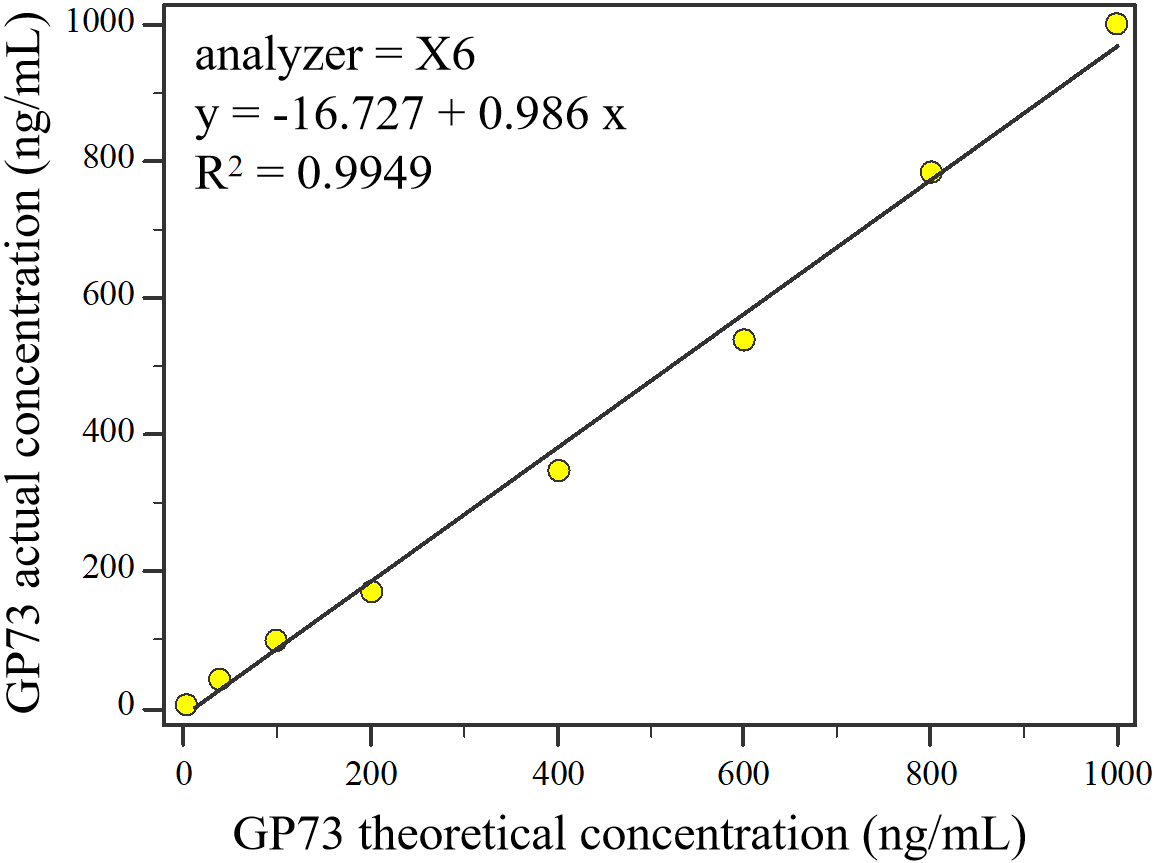

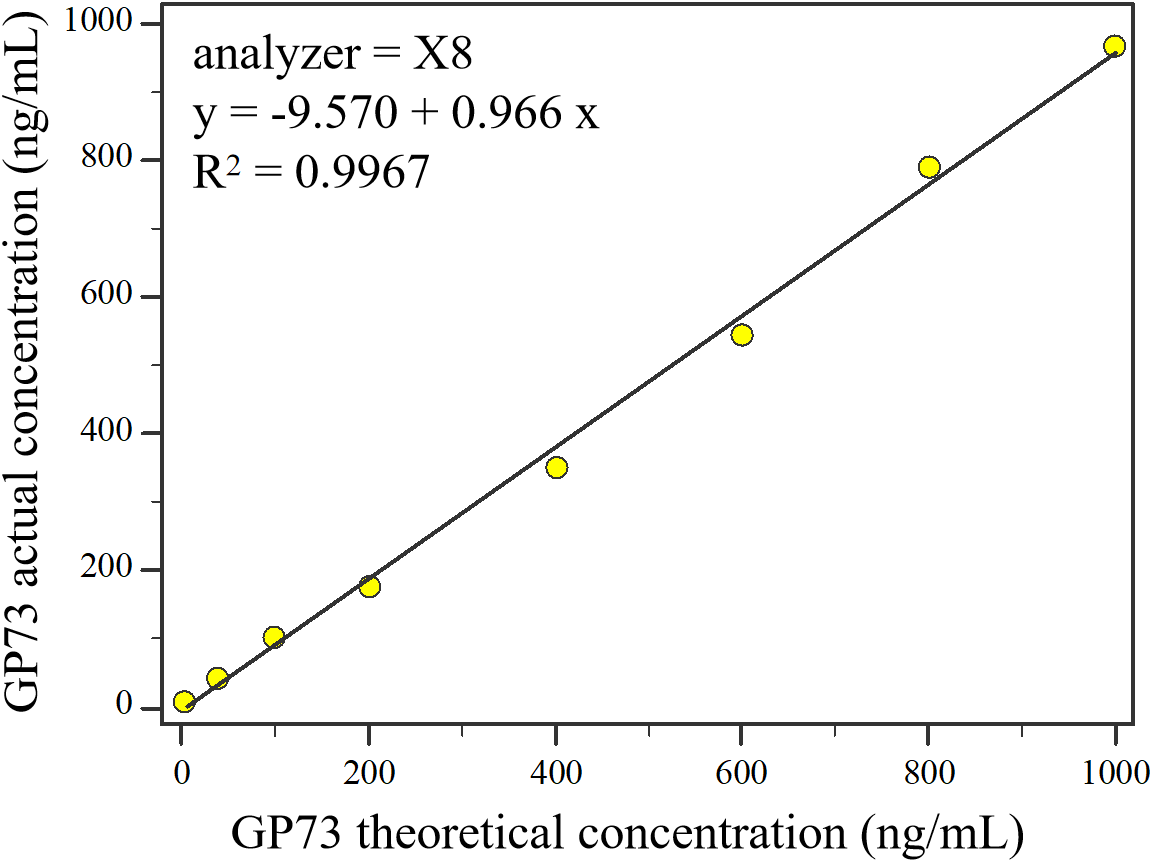


Supplemental Figure 1. Linear correlation analysis of linearity interval test.
